# Comparison of serum apolipoprotein A-I between Chinese multiple sclerosis and other related autoimmune disease

**DOI:** 10.1186/1476-511X-9-34

**Published:** 2010-03-29

**Authors:** Bin Zhang, ShuXiang Pu, BinMei Li, JianRui Ying, Xing Wang Song, Cong Gao

**Affiliations:** 1 Key Laboratory of Neurogenetics and Channelopathies of Guangdong Province and The Ministry of Education of China, The Second Affiliated Hospital of GuangZhou Medical University, 250# Changgang east Road, GuangZhou, 510260 Guangdong Province, China; 2 Institute of Neuroscience, Department of Neurology, The Second Affiliated Hospital of GuangZhou Medical University, 250# Changgang east Road, GuangZhou, 510260 Guangdong Province, China

## Abstract

**Background:**

Serum apolipoprotein (apo) A-I was considered to be an immune regulator and could suppress pro-inflammatory cytokines generated by activated T cell in some autoimmune diseases. However, the change of serum apoA-I levels in multiple sclerosis (MS) patients is unknown.

**Methods:**

In the presentation we performed a study on serum apoA-I levels in the patients with MS. We enrolled some age and gender matched patients with MS, autoimmune demyelinating diseases (Guillain-Barre Syndrome and Clinically Isolated Syndrome), neuroinflammatory diseases (viral encephalitis), autoimmune connective diseases (rheumatoid arthritis and systemic lupus erythematosus) and healthy control groups, and tested their serum lipids levels: total cholesterol (TC), triglyceride (TG), high-density lipoproteins (HDL), apolipoproteinB100 (apoB100), apolipoproteinA-I (apoA-I).

**Results:**

For all patients, age had no effect on serum apoA-I levels (*P *> 0.05). Meanwhile, we proved the highest serum apoA-I levels in MS patients and the lowest serum apoA-I levels in SLE patients. Serum apoA-I levels was significantly elevated in female MS patients (P = 0.033; P < 0.05).

**Conclusion:**

In short we believed that patients with MS and other autoimmune demyelination had significantly decreased serum levels of apo A-I.

## Background

Some previous study suggested that apoA-I was the major structural protein to promote lipid transfer in human plasma, which modulated several cellular functions and involved in the pathogenesis of some autoimmune diseases [1-9]. Hyka et al. approved that apolipoprotein A-I (apo A-I) interfered interreaction between monocytes and activeted T lymphocyte, repressed activation and production of some important pro-inflammatory cytokines in the pathogenesis of some inflammatory and autoimmune diseases (including multiple sclerosis) [6,7].

Multiple sclerosis (MS) is an autoimmune demyelinating disease in central nervous system (CNS) [10,11], and some cytokines secreted by T-help cell (TH1/TH2) play the critical role in initiation and progression of MS [12-14]. Nowadays, more and more study focused on the relationship between apoA-I and autoimmune diseases including rheumatoid arthritis (RA), experimental colitis, thyroiditis and systemic lupus erythematosus (SLE) [15-18]. Although previous studies confirmed elevated serum cholestero, low-density lipoproteins (LDL) and high-density lipoproteins(HDL) during the clinical active phase of experimental allergic encephalomyelitis (EAE) (animal model of MS) [18], few studies explored the effect of apoA-I on MS. Therefore, this is the first study to investigate the relationship between serum apoA-I levels and MS patients.

## Methods

In this clinic-based study, we retrospectively learned 298 hospitalized Chinese patients who had been identified consecutively, examined, treated by our medical staff from January 2002 to July 2008. These patients comprised of 60 Relapsing-Remitting MS patients (mean age, 35.9 ± 14.8 years; female-male, 32:28), 38 patients with Clinically Isolated Syndrome (CIS) including optic neuritis and myelitis (mean age, 36.0 ± 18.3 years; female-male, 19:19; myelitis: optic neuritis, 23:15), 28 patients with Guillain-Barre Syndrome (GBS) (mean age, 36.2 ± 20.0 years; female-male, 10:18), 51 patients with viral encephalitis (mean age, 30.0 ± 13.7 years; female-male, 25:26), 25 patients with rheumatoid arthritis (RA) (mean age, 36.3 ± 9.8 years; female-male, 20:16), 36 patients with systemic lupus erythematosus (SLE) (mean age, 31.6 ± 10.7 years; female-male, 22:14), 60 healthy subjects (mean age, 35.7 ± 10.2 years; female-male, 27:23).

In the presentation, MS patients and RA as well as SLE patients were compared, because research had shown that low serum levels of apoA-I in RA and SLE patients [15,17]. We selected the patients with viral encephalitis in order to compare serum apoA-I levels between the those patients and MS patients. To confirmed the difference between MS patients and other patients with central nervous system autoimmune demyelinating diseases, CIS and GBS patients were selected. Meanwhile, a number of age-matched healthy control group were selected.

All selected patients had never received disease-modifying immunosuppressive therapy that had the affect on plasma lipid or lipoprotein levels two months before admission. All patients were not suffering from diabetes mellitus, liver or thyroid dysfunction, hypertensive disease, cardiovascular disease, stroke, excessive alcohol consumption in their active phase. All MS patients had been diagnose with MS according to the criteria of McDonald et al [19], and scored by the Expanded Disability Status Scale (EDSS) [20]. The mean EDSS score was 3.4 ± 1.99, range 1.0-10. The mean disease course was 5 ± 3.9 years, range 0.1-18 years. All MS patients had the relapsing-remitting (RR) type, RA patients were defined by the 1988 revised criteria of the American College of Rheumatology [21], SLE patients met 1997 criteria for SLE [22].

The blood were collected to detect serum apo A-I at 6 o'clock in the morning and no eating all over the patients and healthy people.

### Statistical analysis

All statistical analyses were performed using the Statistical Program for Social Sciences (SPSS) statistical software (version 11.0, Chicago, IL, USA). Results were expressed as means ± standard deviation (SD). To analyze the effect of age, gender and different entity on serum apoA-I levels in different groups, comparison of serum apoA-I levels among all male or female patients, comparison of serum apoA-I levels between male and female patients in each group using the Multivarite ANOVA. All comparisons were two-sided, with a P-value of less than 0.05 used to indicate statistical significance.

## Results

Table 1 shown age at onset had little effect on serum apoA-I levels, however, different kinds of diseases (P < 0.001) and gender (P < 0.05) have different levels of serum, therefore, we first compared apoA-I levels in different disease groups not taking into account gender and age factors. We found significantly higher serum apoA-I levels in MS (1.392 ± 0.047 g/L) and other autoimmune demyelinating diseases (GBS, CIS) than healthy subjects (1.179 ± 0.047 g/L), RA (1.035 ± 0.061 g/L) and SLE patients(1.179 ± 0.047 g/L). Serum apoA-I levels in RA and SLE patients (P = 0.002) significantly lower than healthy control.

**Table 1:** Analysis of serumapo A-I in the entire patients

Group***	MS	CIS	GBS	Viral encephalitis	SLE	RA	Healthy controls
Gender**(female:male)	32:28	19:19	10:18	26:25	22:14	20:15	27:32
Mean age ± SD*(years)	35.9 ± 14.8	36.0 ± 18.3	36.2 ± 20.0	30.0 ± 13.7	31.6 ± 10.7	36.3 ± 9.8	35.7 ± 10.2
**Apo A-I (g/L)**	1.392 ± 0.047	1.388 ± 0.058	1.282 ± 0.071	1.151 ± 0.051	0.940 ± 0.061	1.03 ± 0.061	1.179 ± 0.047

MS = multiple sclerosis; SLE = systemic lupus erythematosus; RA = rheumatoid arthritis; CIS = Clinically Isolated Syndrome; GBS = Guillain--Barre Syndrome; apoA-I = apolipoproteinA-I.

Data are means ± SD.* No significant differences, multivarite ANOVA, *P *= 0.755(*P *> 0.05). There was no significant effect of age on serum apoA-I levels in different disease groups. ** Significantly different, multivarite ANOVA. *P *= 0.04(*P *< 0.05). There was significant effect of sex on serum apoA-I levels. ***Significantly different, multivarite ANOVA. *P *< 0.001. There was significant effect of gender on serum apoA-I levels.

**Figure 1:**
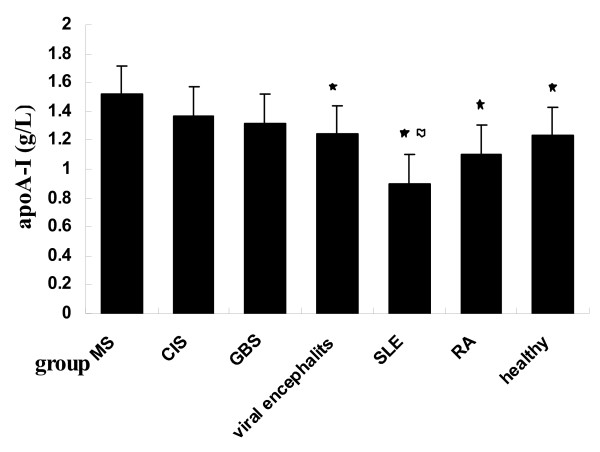
**Comparison of serum apoA- I levels in entire female patients (black star symbol) comparison between MS and other groups, *P *< 0.01; (flag symbol) comparison between healthy controls and SLE patients, *P *< 0.001**.

In order to access the impact of gender on apoA-I, we compared with male and female patients respectively (Table 2). For women, healthy control (1.230 ± 0.062 g/L) had significantly higher serum apoA-I levels than SLE patients (0.897 ± 0.068 g/L; P < 0.001), but significantly lower than female MS patients (1.516 ± 0.057 g/L; P = 0.001). Female patients with viral encephalitis (1.243 ± 0.064 g/L) showed lower serum apoA-I levels than MS patients (P = 0.002). In this study, female SLE patients had the lowest serum apoA-I levels, female MS patients had the highest serum apoA-I levels (Figure 1).

**Table 2:** Analysis of serum apoA-I in all male or female patients.

	MS	CIS	GBS	Viral encephalits	SLE	RA	Healthy controls
**Female**	32	38	28	26	22	20	27
Mean age ± SD*	35.7 ± 14.2	31.2 ± 17.3	29.6 ± 19.9	27.2 ± 13.6	31.4 ± 8.57	35.1 ± 7.75	38.1 ± 14.3
**apoA-I (g/L)**	1.516 ± 0.057	1.368 ± 0.073	1.321 ± 0.101	1.243 ± 0.064	0.897 ± 0.068	1.107 ± 0.072	1.230 ± 0.062
P**	0	0.114	0.097	0.002	0.000	0.000	0.001
**Male**	28	19	10	25	14	15	32
Mean age ± SD*	36.0 ± 15.6	40.7 ± 18.5	39.9 ± 19.7	30.1 ± 13.8	31.9 ± 13.7	37.9 ± 12.1	33.8 ± 4.11
**apoA-I (g/L)**	1.263 ± 0.075	1.422 ± 0.091	1.260 ± 0.093	1.062 ± 0.080	0.979 ± 0.106	0.963 ± 0.102	1.112 ± 0.070
P**	0	0.954	0.194	0.001	0.000	0.000	0.001

Data are means ± SD. * Significant different, multivarite ANOVA, *P *= 0.159 (*P *> 0.05). There was no significant effect of age on female or male serum apoA-I levels in different disease groups. **Compared serum apoA-I levels of between male/female MS patients and male/female patients in other groups, patients with autoimmune demyelinating diseases had higher serum apoA-I levels than other patients and healthy subjects.

**Figure 2:**
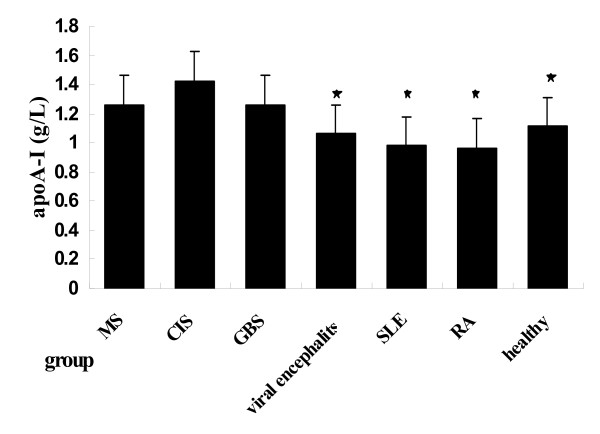
**Comparison of serum apoA- I levels in entire male patients (black star symbol) comparison between MS and other groups, *P *< 0.01**.

For male patients (Table 2), male MS patients (1.263 ± 0.075 g/L) had significantly higher serum apoA-I levels than male RA patients (0.963 ± 0.102 g/L; P = 0.000), male SLE patients (0.979 ± 0.106 g/L; P = 0.000) and male healthy subjects(1.112 ± 0.070 g/L; P = 0.001) (Figure 2). There was no significant different serum apoA-I levels among patients with CIS (1.422 ± 0.091 g/L; P = 0.177), GBS (1.260 ± 0.093 g/L; P = 0.978), viral encephalitis(1.062 ± 0.080 g/L;P = 0.067) and healthy subjects (1.112 ± 0.070 g/L; P = 0.142).

**Figure 3:**
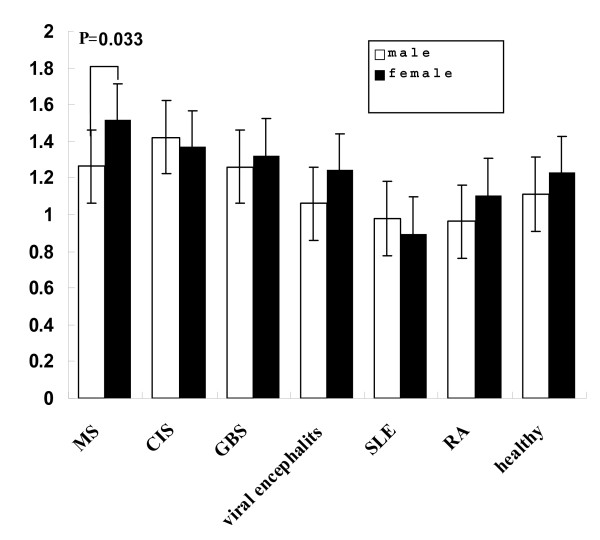
**Comparison of serum apoA- I levels between female and male patients**.

Finally, we compared serum apoA-I between male and female in each disease group (Table 3). The results showed that serum apoA-I levels was much higher in female MS patients (1.523 ± 0.082 g/L) and female RA patients (1.120 ± 0.042 g/L) than the corresponding male MS patients (1.262 ± 0.087 g/L; P = 120.033) and male RA patients (0.948 ± 0.049 g/L; P = 120.012) (Figure 3).

**Table 3:** Analysis of serum apoA-I between male and female patients in different diseases.

Group		Mean age ± SD (years)	apoA-I(g/L)	*P*
**MS**	**Male (n = 28)**	36.0 ± 15.6	1.2620.087	0.033*
	**Female (n = 32)**	35.7 ± 14.2	1.5230.082	
**CIS**	**Male (n = 38)**	40.7 ± 18.5	1.4070.082	0.082
	**Female (n = 19)**	31.2 ± 17.3	1.3700.082	
**GBS**	**Male (n = 28)**	39.9 ± 19.7	1.2650.086	0.876
	**Female (n = 10)**	29.6 ± 19.9	1.2880.116	
**viral encephalitis**	**Male (n = 25)**	30.1 ± 13.8	1.0760.060	0.080
	**Female (n = 26)**	27.2 ± 13.6	1.2210.058	
**SLE**	**Male (n = 14)**	31.9 ± 13.7	0.9840.067	0.296
	**Female (n = 22)**	31.4 ± 8.57	0.8930.053	
**RA**	**Male (n = 15)**	37.9 ± 12.1	0.9480.049	0.012*
	**Female (n = 20)**	35.1 ± 7.75	1.1200.042	
**Healthy controls**	**Male (n = 32)**	33.8 ± 4.11	1.1170.075	0.277
	**Female (n = 27)**	38.1 ± 14.3	1.2410.082	

Data are means ± SD.* Significantly different, multivarite ANOVA, *P *= 0.033 (*P *< 0.05), there was significant effect of gender on female and male serum apoA-I levels in MS patients.

## Discussion

In this study, we found age at onset have a significantly effect on serum apoA-I levels in MS patients relative to other lipid indicators (TG, HDL-C, LDL-C, apoB100), which show that apoA-I is not only associated with serum lipid metabolism, but with the pathogenesis of MS. Shore et al. considered apoA-I was significantly more concentrated during active phase of the EAE (experimental allergic encephalomyelitis, a highly relevant model of MS) than untreated controls [18]. Similar to the Shore ea al, our research showed increased serum apoA-I levels in MS patients and other autoimmune demyelinating disease (CIS, GBS) whether male and female patients. Recently, Gaillard et al conformed that the decreasing CSF (cerebral spinal fluid) apo E (apolipoprotein E) concentrations in MS patients as apoE was postulated to be a major lipid carrier protein [23], therefore, we wonder if the CSF apoA-I concentrations would be increased in MS patients, our next task is to conform the hypothesis.

The imbalance between pro-inflammatory cytokines and anti-inflammatory cytokines would lead to autoimmune diseases such as RA, MS, SLE, atherosclerosis [24-27], these cytokines production were modulaed by contact-mediated induction between monocytes and stimulated T lymphocyte. ApoA-I bound the stimulating factor at the surface of T lymphocytes, hampered the binding of stimulated T lymphocytes with its specific receptor at monocyte surface, thus inhibited the production of pro-inflammatory cytokines including TNF-α and IL-1 [6,28]. Therefore, some researchers believed serum apoA-I concentrations should be declined during active phase of autoimmune diseases, and has played an important role in anti-inflammation, such as RA, SLE [29-31]. Consisted with above findings, in our study, serum apoA-I levels in RA and SLE patients were significantly lower than healthy subjects. It is interesting that MS patients had the highest serum apoA-I levels contrary to the hypothesis of above studies.

The reason remained unknown, but some emerging evidence that may explain this phenomenon. Some reports considered serum apoA-I was an inhibitory factor as a "negative" acute-phase protein, they suggested that apoA-I might be transported and get into the "leaky" blood-brain barriers by cerebral endothelial cells, and proposed apoA-I could enter the demyelinating nerve to regenerate impaired nerve and myelin from plasma when the blood-nerve barrier was disrupted after injury [32,33]. In recent years, some researchers conformed that astrocytes generated apoA-I and apoE in rat, apoA-I facilitated translocation of newly synthesized cholesterol and phospholipid to cytosol to form the lipid-protein complex particles as an initial event in cholesterol trafficking for the assembly of HDL, and found cholesterol efflux from rat astrocytes induced by apoA-I and apoE. In CNS, apoA-I could modulate transport of cholesterol and reduce CNS impairments by activating the brain lecithin cholesterol acyl transferase (LCAT) [34-36]. They found apoA-I had increased 26-fold in rat homogenates of regenerating sciatic nerves within 3 weeks after injury [35]. Therefore, a large number of serum apoA-I synthesized by liver will be albe to meet the remyelination during acute phase of MS.

In the study, our data showed elevated serum apoA-I concentrations in MS patients may be an important feature that is different from other autoimmune diseases which had significantly reduced serum apoA-I levels (such as RA and SLE). In order to clarify the effect of CNS inflammatory response on serum apoA-I levels, we compare neuroinflammation patients with MS patients, This study further confirmed that, compared to other CNS inflammatory diseases, the imbalance between demyelination and regeneration in MS patients may be related to elevated serum apoA-I concentrations.

Finally, the results indicated that female MS patients had significant higher serum apoA-I levels than male MS patients, but this phenomenon have not been found in other demyelinating diseases. The reason remained unknown, but it may be associated with the greater susceptibility and incidence of female MS patients.

## Conclusion

MS patients had the highest serum apoA-I levels compared with other disease groups and healthy control, and female MS patients had a significant higher levels than male MS patients. Then the following work should be done to expose the reason for our results, determine the CSF apoA-I levels in MS patients and discuss relationship between serum/CSF apoA-I and anti-inflammatory cytokines.

## Competing interests

The authors declare that they have no competing interests.

## Authors' contributions

BZ and CG, co-designed and coordinated the study as well as prepared; BZ carried out lipids measurements, analytical work; BZ and SP carried out analytical and statistical; JY carried out analytical work. All authors read and approved the final manuscript.

## References

[B1] Koner BC Goswami K Kavitha S Moorthy RS Normal lipid metabolism, familial hyperlipidaemia, lipid intervention and theirbenefits J Indian Med Assoc 2003 101 2 89 92 Review 12841490

[B2] Remaley AT Thomas F Stonik JA Synthetic amphipathic helical peptides promote lipid efflux from cells by an ABCA1-dependent and an ABCA1-independent pathway J Lipid Res 2003 44 828 36 10.1194/jlr.M200475-JLR200 12562845

[B3] Shah PK Yano J Reyes O High-dose recombinant apolipoproteinA-I(milano) mobilizes tissue cholesterol and rapidly reduces plaque lipid and macrophage content in apolipoprotein e-deficient mice. Potential implications for acute plaque stabilization Circulation 2001 103 3047 50 10.1161/hc2501.092494 11425766

[B4] Martinez LO Agerholm-Larsen B Wang N Chen W Tall AR Phosphorylation of a pest sequence in ABCA1 promotes calpain degradation and is reversed by ApoA-I Biol Chem 2003 278 37368 74 10.1074/jbc.M307161200 12869555

[B5] Zhang Y Zanotti I Reilly MP Glick JM Rothblat GH Rader DJ Overexpression of apolipoprotein A-I promotes reverse transport of cholesterol from macrophages to feces in vivo Circulation 2003 108 661 63 10.1161/01.CIR.0000086981.09834.E0 12900335

[B6] Burger D Dayer JM High-density lipoprotein-associated apolipoprotein A-I: the missing link between infection and chronic inflammation? Autoimmun Rev 2002 1 111 7 10.1016/S1568-9972(01)00018-0 12849067

[B7] Hyka N Dayer JM Modoux C Kohno T Edwards C K III Apolipoprotein A-I inhibits the production of interleukin-1b and tumor necrosis factor-a by blocking contact-mediated activation of monocytes by T lymphocytes Blood 2001 97 2381 8 10.1182/blood.V97.8.2381 11290601

[B8] Cigliano L Spagnuoloa MS Cuomo G Apolipoprotein A-I-dependent cholesterol esterification in patients with rheumatoid arthritis Life Sci 2005 77 108 20 10.1016/j.lfs.2004.12.023 15848223

[B9] Bairaktari E Tselepis AD Millionis HJ Elisaf MS Lipoprotein (a) levels, apolipoprotein(a) phenotypes and thyroid autoimmunity Eur J Endocrinol 1999 140 474 6 10.1530/eje.0.1400474 10229916

[B10] El Behi M Dubucquoi S Lefranc D New insights into responses involved in experimental autoimmune encephalomyelitis and multiple sclerosis Immunol Lett 2005 96 11 26 10.1016/j.imlet.2004.07.017 15585303

[B11] Zappulla JP Arock M Mars LT Mast cells: new targets for multiple sclerosis therapy? J Neuroimmunol 2002 131 5 20 10.1016/S0165-5728(02)00250-3 12458032

[B12] Desplat-Jégo S Creidy R Varriale S Allaire N Anti-TWEAK monoclonal antibodies reduce immune cell infiltration in the central nervous system and severity of experimental autoimmune encephalomyelitis Clin Immunol 2005 117 15 25 10.1016/j.clim.2005.06.005 16027043

[B13] Hilliard B Wilmen A Seidel C Liu TS Roles of TNF-Related Apoptosis-Inducing Ligand in Experimental Autoimmune Encephalomyelitis J Immunol 2001 166 1314 19 11145715 10.4049/jimmunol.166.2.1314

[B14] Campbell S Burkly LC Gao HX Berman JW Proinflammatory Effects of Tweak/Fn14 Interactions in Glomerular Mesangial Cells J Immunol 2006 176 1889 98 16424220 10.4049/jimmunol.176.3.1889

[B15] Rossol M Kaltenhäuser S Scholz R Thecontact-mediated response of peripheral-blood monocytes to preactivated T cells is suppressed by serum factors in rheumatoid arthritis Arthritis Res Ther 2005 7 1189 99 10.1186/ar1804 PMC1297564 16277671

[B16] Vowinkel T Mori M Krieglstein C Apolipoprotein A-IV inhibits experimental colitis J Clin Invest 2004 114 260 9 15254593 10.1172/JCI21233 PMC450164

[B17] Dinu AR Merrill JT Shen C Frequency of antibodies to the cholesterol transport protein apolipoprotein A1 in patients with SLE Lupus 1998 7 5 355 360 10.1191/096120398678920262 9696140

[B18] Shore VG Smith ME Perret V Laskaris MA Alterations in plasma lipoproteins and apolipoproteins in experimental allergic encephalomyelitis J Lipid Res 1987 28 119 29 3494804

[B19] McDonald W Compston A Edan G Recommended diagnostic criteria for multiple sclerosis: guidelines from the international panel on the diagnosis of multiple sclerosis Ann Neurol 2001 50 121 7 10.1002/ana.1032 11456302

[B20] Kurtzke JF Rating neurologic impairment in multiple sclerosis: an expanded disability status scale (EDSS) Neurology 1983 33 1444 52 6685237 10.1212/wnl.33.11.1444

[B21] Arnett FC Edworthy SM Bloch DA McShane DJ Fries JF Cooper NS Healey LA Kaplan SR Liang MH Luthra HS The American Rheumatism Association 1987 revised criteria for the classification of rheumatoid arthritis Arthritis Rheum 1988 31 315 24 10.1002/art.1780310302 3358796

[B22] Hochberg MC Updating the American College of Rheumatology revised criteria for the classification of systemic lupus erythematosus Arthritis Rheum 1997 40 1725 10.1002/art.1780400928 9324032

[B23] Gaillard O Gervai A Meillet D Plassart E Apolipoprotein E and multiple sclerosis: A biochemical and genetic investigation J Neurol Sci 1998 158 180 86 10.1016/S0022-510X(98)00118-X 9702689

[B24] Burger D Dayer JM Inhibitory cytokines and cytokine inhibitors Neurology 1995 45 39 43 10.1212/wnl.45.6_suppl_6.s39 7783910

[B25] Feldmann M Brennan FM Maini RN Role of cytokines in rheumatoid arthritis Annu Rev Immunol 1996 14 397 440 10.1146/annurev.immunol.14.1.397 8717520

[B26] Lucas K Hohlfeld R Differential aspects of cytokines in the immunopathology of multiple sclerosis Neurology 1995 45 4 5 10.1212/wnl.45.6_suppl_6.s4 7783911

[B27] Sullivan GW Sarembock IJ Linden J The role of inflammation in vascular diseases J Leukoc Biol 2000 67 591 602 10810997 10.1002/jlb.67.5.591

[B28] Gennaro DL Lucia M How T lymphocytes recognize lipid antigens FEBS Letters 2006 580 5580 87 10.1016/j.febslet.2006.08.029 16949584

[B29] Barry B Martina G Oliver FG Jean MD Apolipoprotein A-I infiltration in rheumatoid arthritis synovial tissue: a control mechanism of cytokine production? Arthritis Res Ther 2004 6 563 66 10.1186/ar1443 PMC1064871 15540281

[B30] Abe H Tsuboi N Suzuki S Sakuraba H Anti-apolipoprotein A-I autoantibody: characterization of monoclonal autoantibodies from patients with systemic lupus erythematosus Rheumatol 2001 28 5 990 95 11361227

[B31] McMahon M Grossman J Chen W Hahn BH Inflammation and the pathogenesis of atherosclerosis in systemic lupus erythematosus Lupus 2006 15 59 69 10.1177/096120330607166816482750

[B32] Gabay C Kushner I Acute-phase proteins and other systemic responses to inflammation N Engl J Med 1999 340 448 54 10.1056/NEJM199902113400607 9971870

[B33] Janet KB Lucia MN Linda J Anderson. Accumulation of Apolipoproteins in the Regenerating Remyelinating Mammalian Peripheral Nerve J Biol Chem 1990 265 17805 15 2120218

[B34] De Vries HE Breedveld B Kuiper J de Boer AG Van Berkel TJ Breimer DD High-density lipoprotein and cerebral endothelial cells in vitro: interactions and transport Biochem Pharmaco 1995 50 271 3 10.1016/0006-2952(95)00127-L 7632172

[B35] Demeester G Castro C Desrumaux CD Characterization and functional studies of lipoproteins, lipid transfer proteins, and lecithin cholesterol acyl transferase in CSF of normal individuals and patients with Alzheimer's disease J Lipid Res 2000 41 963 74 10828089

[B36] Jin-ichi Ito Yuko Nagayasu Koichi Kato Ryuichiro Sato ApolipoproteinA-I Induces Translocation of Cholesterol, Phospholipid, and Caveolin-1 to Cytosol in Rat Astrocytes J Biol Chem 2002 277 7929 35 10.1074/jbc.M103878200 11773045

